# Vitamin D status and distribution in patients with chronic obstructive pulmonary disease versus healthy controls

**Published:** 2015

**Authors:** Behzad Heidari, Yahya Javadian, Mahmoud Monadi, Yahya Dankob, Alireza Firouzjahi

**Affiliations:** 1 Department of Internal Medicine, Rouhani Hospital, Babol University of Medical Sciences, Babol, Iran.; 2 Mobility Impairment Research Center, Babol University of Medical Sciences Babol, Iran.; 3 Department of Laboratory Medicine, Ayatollah Rouhani Hospital, Babol University of Medical Sciences, Babol, Iran.

**Keywords:** Vitamin D, Chronic obstructive pulmonary disease, Deficiency, Insufficiency

## Abstract

**Background:**

Vitamin D has a potential to modulate inflammatory response against noxious particles in patients with chronic obstructive pulmonary disease (COPD). The present study was conducted to determine the status of serum vitamin D in COPD versus healthy group.

**Methods:**

The patients presented to the outpatient pulmonary clinic of Ayatollah Rouhani Hospital, Babol Iran. Diagnosis of COPD was confirmed based on airflow limitation defined as FEV1/FVC ratio <70% and FEV1< 80% of predicted. All eligible patients aged ≥ 40 years old entered the study. Pulmonary infection, tuberculosis, pleural effusion, congestive heart failure, pulmonary hypertension and embolism, restrictive airway disease, conditions leading changes in vitamin D metabolism and absorption were excluded. Serum 25-hydroxyvitamin D (25-OHD) was determined by electrocheminluminescence method and levels <20, 20-29, and ≥30ng/ml were considered as deficiency, insufficiency, and sufficiency. In statistical analysis, the frequency of serum 25-OHD deficiency and insufficiency in patients were compared regarding age of ≤ 50 or >50 years old. All patients were males and age and sex-matched controls were selected among healthy subjects accompanied COPD patients.

**Results:**

Ninety patients and 100 controls with respective mean (±SD) age of 64.8±11.7 and 62.6±11.7 years old (P=0.19) were studied. Compared with control, proportions of serum 25-OHD deficiency and insufficiency in patients >50 years were higher and deficiency was lower (61.5% vs 87.5%, P=0.11).

**Conclusion:**

These findings indicate that a significant proportion of young COPD patients have insufficient serum 25-OHD. Regarding a positive relationship between 25-OHD and FEV1 in COPD, these findings highlight serum 25-OHD assessment in COPD for recognizing high risk patients.

Vitamin D deficiency is a problem which affects a substantial proportion of general population around the world (1, 2). It is linked with the development as well as the progression of several conditions including skeletal and non-skeletal diseases (2-8). The impacts of vitamin D deficiency have been addressed in several diseases like autoimmune diseases, chronic obstructive pulmonary disease (COPD) and cancer (8). It is believed that patients with pulmonary diseases such as COPD are at greater risk of vitamin D deficiency (9) and the prevalence of deficiency increases with the severity of disease stage (10, 11). It has been shown that insufficient vitamin D may result in low lung function during exposure to environmental agents (12). 

The relationship between vitamin D and pulmonary function was shown (13-16). In addition, vitamin D deficient patients with COPD have greater predisposition to respiratory infections and COPD exacerbations (17, 18). Airway inflammation is the main cause of obstruction in COPD. 

Inflammation in COPD has been attributed to inflammatory responses to noxious particles and gases resulting in persistent airflow obstruction (19, 20).

Vitamin D suppresses airway inflammation and prevents allergic asthma in mice (8, 21). Airway remodeling which is the most characteristic pathogenic hallmark of COPD can be affected by vitamin D deficiency (12).These observations indicate that sufficient levels of serum vitamin D in patients with COPD may impose COPD patients to higher risk of respiratory infection or COPD exacerbations. Data regarding vitamin D and COPD are not adequate and the results of previous studies are conflicting. For these reasons, the present study was performed to determine the status of serum vitamin D in patients with COPD in comparison with healthy controls.

## Methods

**Study population: **The study patients of this case-control study were derived from COPD patients presenting to outpatient pulmonary clinic of the Ayatollah Rouhani Hospital, a university affiliated teaching hospital in Babol, North of Iran. The diagnosis of COPD was confirmed with compatible clinical features concurrent with airflow limitation defined as FEV1/ forced vital capacity (FVC) less than 0.70 (FEV1/FVC ratio <70%) and FEV1< 80% predicted (22).

 All patients were selected prospectively according to inclusion criteria (16). All eligible cases aged ≥ 40 years old entered to the study. Exclusion criteria included, presence of pulmonary infection, tuberculosis, pleural effusion, congestive heart failure, primary pulmonary hypertension, pulmonary emboli, restrictive airway disease, conditions associated with altered vitamin D metabolism and abnormal absorption of vitamin D, taking vitamin D, and oral corticosteroids. All selected patients were men. Age and sex-matched controls were selected among healthy accompanying of the patients presented to the same hospital. A similar exclusion criterion as used for patients was also applied for the control group.

**Data collection: **Data were collected through an interview in regard to age, previous illness, medications such as beta agonist and anticholinergic bronchodilators, inhaled corticosteroids, weight, smoking and opium addiction. Serum vitamin D was assessed by quantitative determination of 25-hydroxyvitamin D (25-OHD) by electrocheminluminescence method using Elecsys vitamin D total reagent according to the manufacturer’s instruction. Serum 25-OHD concentrations of less than 20ng/ml were considered as deficiency, 20-29 ng/ml insufficiency and 30 ng/ml and more sufficiency (2). Sample size was estimated to determine 20% difference in vitamin D deficiency between patients with COPD and controls with 80% power of statistical test and confidence interval of 95%.

**Pulmonary function tests: **Post bronchodilator FEV1, FEV1% was assessed at the time of data collection. All tests were performed by a single experienced technician in the hospital. 

**Statistical analysis: **In statistical analysis, serum 25-OHD status (serum 25-OHD concentration and frequency of patients and controls with deficiency and insufficiency) were determined and compared. Mann-Whitney U test was used for comparing serum 25-OHD levels between the two groups and chi square test was used for the comparison of proportions. Additional analysis was performed to determine whether serum vitamin D status differs between patients aged >50 years old and ≤50 years old as observed in our earlier study (5). The proposal of this study was approved by the Ethics Committee of Babol University of Medical Sciences, Babol, Iran. 

## Results

Ninety patients with mean (±SD) age of 64.8±11.7 years old and 100 controls with mean age (±SD) 62.6±11.7 years old (P=0.19) entered the study. Mean FEV1% and FEV1 volume in patients was 60.13±13.5% and 1728±639 ml, respectively. Serum 25-OHD distribution was skewed to the right in patients with median value of 25 (9.5-57) ng/ml and in the control 23(4-185) ng/ml (P=0.013).

Proportions of serum 25-OHD deficiency, insufficiency and sufficiency varied across the two groups of patients and controls (table 1). However, distribution of serum 25-OHD in patients was slightly skewed to the right whereas; in the control group distribution was severely right skewed. So, serum 25-OHD deficiency was seen in 14 (15.6%) patients but in 46(46%) controls (P=0.001). In contrast, insufficient serum 25-OHD in patients was significantly higher than controls (51.1% vs 20%, P=0.001). Frequency of serum 25-OHD deficiency and insufficiency was comparable between the patients and controls and did not differ significantly with regard to age of ≤50 and >50 years old (table 2).

**Table 1:** Frequency of serum 25-OHD deficiency insufficiency and sufficiency in patients with chronic obstructive pulmonary disease versus controls

**Serum 25-OHD level**	**Patients N (%)**	**Controls N (%)**	**P values **
< 20 ng/ml	14(15.6)	46(46)	0.001
20-29 ng/ml	46(51.1)	20(20.)	0.001
≥30 ng/ ml	30(33.3)	34(34)	0.52

**Table 2:** Frequency of serum 25-hydroxyvitamin D (25-OHD) deficiency (<20ng/ml), insufficiency (20-29ng/ml) and sufficiency (≥30ng/ml) in patients with chronic obstructive pulmonary disease (COPD) versus healthy control according to age of ≤50 and >50 years

**Serum 25-OHD status**	**Controls(n=100)**		**Patients (n=90)**	
	**≤50 years ** **n(%)**	**>50 years** **n(%)**	**≤50 years ** **n(%)**	**>50 years ** **n(%)**
< 20ng/ml	11 (68.8)	35 (41.7)	1 (7.7)	13 (16.9)
20-29ng/ml	3 (18.7)	17 (20.2)	7 (53.8)	39 (50.6)
>30ng/ml	2 (12.5)	32 (38.1)	5 (38.5)	25 (32.5)
Total	16	84	13	77

## Discussion

 The results of this study indicated that vitamin D insufficiency is common in COPD and a significant proportion of these patients have vitamin D deficiency or insufficiency regardless of age. Serum 25-OHD status in the study population was comparable to control indicating reflection of vitamin D status in the general population. However, distribution of serum 25-OHD in COPD differs significantly from the control group. Thus, serum 25-OHD deficiency was less prevalent in COPD patients, whereas serum insufficiency was more prevalent in the control group.

 In the present study, more than 50% of patients with COPD had insufficient levels of serum 25-OHD which is in agreement with the results of earlier studies (23-25). Low serum 25-OHD in COPD is important, because there is a dose- response relationship between serum vitamin D and FEV1 (13, 16), Black et al. have shown a positive dose-response relationship between serum 25-OHD and FEV1 in patients of the Third National Health and Nutrition Examination Survey (13). Even in healthy subjects pulmonary function was shown to be related with serum 25-OHD levels or daily intake of vitamin D (14). Mahlin et al. showed low vitamin D diet and low serum vitamin D in both COPD and the elderly population of Sweden (26 ).In another cross-sectional study, there was an inverse association between serum 25-OHD and COPD (27).On the other hand, a study found no association of vitamin D and COPD phenotypes (28).

The patients with insufficient serum 25-OHD are expected to have lower FEV1 and rising serum 25-OHD to sufficient levels may improve pulmonary function as well as host defense (14, 16, 29). Efficiency of vitamin D supplementation on pulmonary function in COPD was shown in our previous study (30). Insufficient serum 25-OHD imposes COPD patients to greater risk of upper respiratory infections and COPD exacerbation (17, 18).

 These observations justify the correction of serum 25-OHD to sufficient level in COPD. Raising serum vitamin D to sufficient levels exerts additional advantages on extra pulmonary complications of COPD (8). In particular, osteoporosis and muscle weakness are prevalent in COPD (25). Serum 25-OHD correlates positively with bone mineral density (31). Vitamin D supplementation increases bone density and improves inspiratory muscle strength, and muscle function and oxygen uptake during pulmonary rehabilitation (32, 33). The results of this study should be considered with limitations. We did not provide data because of vitamin D intake and sunlight exposure and physical activity in our patients and control. Low physical activity, indoor activity, and sunlight deprivation are common in COPD patients, and these factors may have contribution in the development of serum 25-OHD deficiency and so affect the results. However, we have found no seasonal variations in serum vitamin D in this geographic region (34). Irrespective to etiology of vitamin D deficiency, optimal levels of serum vitamin D are expected to be associated with higher pulmonary function and lower extra pulmonary complications.

## Conclusion

The findings of this study indicate vitamin D insufficiency in a significant proportion of patients with COPD which reflects vitamin D status of the general population. Regarding several pulmonary and extra pulmonary adverse effects of vitamin D deficiency, these findings justify serum vitamin D assessment in all patients with COPD and raising it to optimal level.

## Notes

**Funding:** This study was funded by the Vice-Chancellery for Research and Technology of Babol University of Medical Sciences, Babol, Iran
